# Identification of Novel Long Noncoding RNAs and Their Role in Abdominal Aortic Aneurysm

**DOI:** 10.1155/2020/3502518

**Published:** 2020-12-21

**Authors:** Abulaihaiti Maitiseyiti, Hongbo Ci, Qingbo Fang, Sheng Guan, Alimujiang Shawuti, Huguo Wang, Xiaohu Ge

**Affiliations:** ^1^Xinjiang Medical University, Urumqi, Xinjiang Uygur Autonomous Region, China; ^2^Department of Vascular Surgery, People's Hospital of Xinjiang Uygur Autonomous Region, Urumqi, Xinjiang Uygur Autonomous Region, China; ^3^Department of Vascular and Thyroid Surgery, Center of Digestive and Vascular Surgery, The First Affiliated Hospital of Xinjiang Medical University, Urumqi, Xinjiang Uygur Autonomous Region, China

## Abstract

**Objective:**

Long noncoding RNAs (lncRNAs) have emerged as critical molecular regulators in various diseases. However, the potential regulatory role of lncRNAs in the pathogenesis of abdominal aortic aneurysm (AAA) remains elusive. The aim of this study was to identify crucial lncRNAs associated with human AAA by comparing the lncRNA and mRNA expression profiles of patients with AAA with those of control individuals.

**Materials and Methods:**

The expression profiles of lncRNAs and mRNAs were analyzed in five dilated aortic samples from AAA patients and three normal aortic samples from control individuals using microarray technology. Functional annotation of the screened lncRNAs based on the differentially expressed genes was performed using Gene Ontology (GO) and Kyoto Encyclopedia of Genes and Genomes (KEGG) analyses.

**Results:**

Microarray results revealed 2046 lncRNAs and 1363 mRNAs. Functional enrichment analysis showed that the mRNAs significantly associated with AAA were enriched in the NOD-like receptor (NLR) and nuclear factor kappa-B (NF-*κ*B) signaling pathways and in cell adhesion molecules (CAMs), which are closely associated with pathophysiological changes in AAA. The lncRNAs identified using microarray analysis were further validated using quantitative real-time polymerase chain reaction (qRT-PCR) analysis with 12 versus 11 aortic samples. Finally, three key lncRNAs (ENST00000566954, ENST00000580897, and T181556) were confirmed using strict validation. A coding-noncoding coexpression (CNC) network and a competing endogenous RNA (ceRNA) network were constructed to determine the interaction among the lncRNAs, microRNAs, and mRNAs based on the confirmed lncRNAs.

**Conclusions:**

Our microarray profiling analysis and validation of significantly expressed lncRNAs between patients with AAA and control group individuals may provide new diagnostic biomarkers for AAA. The underlying regulatory mechanisms of the confirmed lncRNAs in AAA pathogenesis need to be determined using in vitro and in vivo experiments.

## 1. Introduction

Abdominal aortic aneurysm (AAA) is a common, potentially fatal cardiovascular disease characterized by weakening of the local aortic wall, followed by progressive expansion and eventually rupture of the dilated aortic segment [1]. AAA is generally asymptomatic and occurs in up to 8% of men over the age of 65 years and 1.53% of women over the age of 60 years [2]. The approximately 13000 deaths annually attributed to AAA rupture in the United States are underestimated [3]. Currently, the only available treatment option remains open or endovascular surgery [4]. To date, there are no effective drugs that can limit the growth of aneurysms and consequently prevent aortic rupture. However, the detailed mechanisms of AAA progression and the nature of aortic rupture are not fully understood [5]. The hallmark pathological features that lead to AAA formation are complex, including phenotype switching of vascular smooth muscle cells (VSMCs), VSMC apoptosis, extensive infiltration of inflammatory cells, extracellular matrix (ECM) remodeling, intense oxidative stress, endothelial cell (EC) dysfunction, intraluminal thrombus formation, and progressive deterioration of the aortic wall [6].

Long noncoding RNAs (lncRNAs) are the largest class of ncRNAs and are defined as transcribed RNA molecules > 200 nucleotides in length and without significant protein-coding potential [7]. lncRNAs have the potential to regulate the expression of genes at the epigenetic, transcriptional, and posttranscriptional levels and play an important role in physiological processes [8]. lncRNA expression and regulation can be highly tissue-, cell-, and disease context-specific, which makes them promising therapeutic intervention and biomarker candidates [9, 10]. lncRNA microarray technology is a new tool that can be used to explore the crucial genes involved in several diseases, reveal diagnostic biomarkers, and the underlying molecular mechanisms [11].

Several studies have shown that lncRNAs play an important role in the pathogenesis of cardiovascular disease and that they could function as disease biomarkers. This was demonstrated in previous studies, such as that of Kumarswamy et al. [12], which revealed that long noncoding RNA uc022bqs.1 (LIPCAR) was downregulated in the early stages after myocardial infarction but upregulated during the later stages, which indicated that LIPCAR is a novel biomarker of cardiac remodeling and that it can predict future death in patients with heart failure. Xu et al. [13] reported that lnc-TNFSF14 is a potential target regulating matrix degradation in type B aortic dissection development. Numerous previous microarray analyses have been performed to screen AAA-related lncRNAs in mouse tissue, and microarray-based methods have identified lncRNAs without validating them in large sample sizes. However, little is known about lncRNA-related biomarkers and their regulatory role in AAA progression.

In this study, we identified differentially expressed lncRNAs between the AAA and control groups. Moreover, the initially identified lncRNAs were validated using q-PCR in a large number of samples. Finally, functional annotation was performed and related pathways were predicted based on the differentially expressed genes. A regulatory network was constructed based on these confirmed lncRNAs. Our newly discovered lncRNAs may provide new biomarker candidates and therapeutic targets for AAA studies in the future.

## 2. Materials and Methods

### 2.1. Patients and Sample Collection

The diagnostic criteria of AAA were based on the Society for Vascular Surgery practice guidelines on the care of patients with an abdominal aortic aneurysm [14]. Five dilated abdominal aortic samples from patients with AAA and three normal aortic samples from control subjects obtained during the operation were used in the lncRNA microarray analysis. The results were validated in the same original samples using PCR. The hub genes (lncRNAs) were further confirmed in 11 dilated aortic samples from patients with AAA and 12 normal aortic samples from control individuals using PCR. Full-thickness aortic wall samples were collected from the anterior wall of the infrarenal AAA (aneurysmal area) of patients who underwent elective open AAA repair. Meanwhile, normal aortic samples from the ascending aorta without visible atherosclerotic changes were collected from patients undergoing aortic valve replacement surgery. AAA and normal aorta tissues were snap-frozen in liquid nitrogen immediately after resection and then transferred to -80°C until use. This study was approved by the ethical committee of the People's Hospital of Xinjiang Uygur Autonomous Region. All the patients who participated in the study provided written informed consent before entering the study. This study was performed in accordance with the World Medical Association Declaration of Helsinki.

### 2.2. RNA Extraction and Quality Control

The TRIzol® reagent (Invitrogen, Carlsbad, CA, USA) was used to extract total RNA according to the manufacturer's instructions. In brief, approximately 20 mg of prefrozen aortic wall tissue was homogenized using the Mini-Bead-Beater-16. RNA phase separation and precipitation were performed using chloroform and isopropyl alcohol, respectively. Then, RNA was washed with 75% ethanol and dissolved in RNase-free water. A NanoDrop ND-1000 (Thermo Fisher Scientific) was used to evaluate the concentration and purity of total RNA. The integrity of the RNA was assessed using standard denaturing agarose gel electrophoresis.

### 2.3. lncRNA and mRNA Microarray Analysis

The Arraystar Human LncRNA Microarray V4.0 is designed for the global profiling of human lncRNAs and protein-coding transcripts, and approximately 40173 lncRNAs and 20730 coding transcripts can be detected using a third-generation lncRNA microarray. We used the Arraystar Human LncRNA Microarray V4.0, which was designed to detect the differential expression of lncRNAs and mRNAs between the AAA and control groups. Sample labeling and array hybridization were performed according to the Agilent One-Color Microarray-Based Gene Expression Analysis protocol (Agilent Technology) with minor modifications. Briefly, mRNA was purified from total RNA after removal of rRNA (mRNA-ONLY™ Eukaryotic mRNA Isolation Kit, Epicentre). Then, each sample was amplified and transcribed into fluorescent cRNA along the entire length of the transcripts without 3′ bias using a random priming method (Arraystar Flash RNA Labeling Kit, Arraystar). The labeled cRNAs were purified using the RNeasy Mini Kit (Qiagen). The concentration and specific activity of the labeled cRNAs (pmol Cy3/*μ*g cRNA) were measured using the NanoDrop ND-1000. One microgram of each labeled cRNA was fragmented by adding 5 *μ*L 10× blocking agent and 1 *μ*L of 25× fragmentation buffer and heating the mixture at 60°C for 30 min. Finally, 25 *μ*L 2× GE hybridization buffer was added to dilute the labeled cRNA. A total of 50 *μ*L of hybridization solution was dispensed into the gasket slide and assembled onto the lncRNA expression microarray slide. The slides were incubated for 17 h at 65°C in an Agilent Hybridization Oven. The hybridized arrays were washed, fixed, and scanned using the Agilent DNA Microarray Scanner (part number G2505C).

### 2.4. Gene Ontology (GO) and Kyoto Encyclopedia of Genes and Genomes (KEGG) Pathway Analyses

GO and KEGG pathway analyses were used to investigate the functions of differentially expressed mRNAs. GO analysis contained biological processes (BP), molecular functions (MF), and cellular components (CC). Differentially expressed mRNAs were classified into different GO terms that described gene functions and attributes based on the GO database (http://www.geneontology.org/). A biological pathway analysis based on the KEGG database (http://www.genome.jp/kegg/) was performed to determine the biological pathways for the enrichment of expressed mRNAs associated with differently expressed lncRNAs. Statistically significant GO terms and pathways were identified using Fisher's exact and chi-square tests. The threshold of significance was defined as a *P* of <0.05, and the false discovery rate (FDR) was calculated to correct the *P* value.

### 2.5. qRT-PCR Validation Assay

To confirm the reliability of the microarray data, the expression levels of the lncRNAs selected in the microarray and preliminary PCR screening stages were further validated in a large number of samples. Total RNA isolated from aortic samples was extracted using the TRIzol reagent. Reverse transcription was performed after RNA quantification, using a reverse transcription PCR kit following the manufacturer's instructions (Takara, Kusatsu, Japan). Real-time PCR was performed using the SYBR quantitative real-time PCR kit (Takara) on an ABI Prism 7500HT instrument (Applied Biosystems, Foster City, CA). Each sample was measured in triplicate, and the mean value was used for comparative analysis. The relative expression of lncRNAs was calculated using the 2^-*ΔΔ*Ct^ method and normalized to the expression of *β*-actin.

### 2.6. Construction of the lncRNA-mRNA Coding-Noncoding Coexpression (CNC) Network

A lncRNA-mRNA CNC was used to determine the interactions between the differentially expressed lncRNA and mRNA groups. The relevance of each lncRNA-mRNA pair was calculated using Pearson's correlation coefficient (PCC) with coefficients not less than 0.97, *P* value ≤ 0.05, and FDR ≤ 1 between the mRNAs and the lncRNAs. lncRNA-mRNA pairs that met these criteria were chosen to construct this network using the Cytoscape software (version 3.7.1, The Cytoscape Consortium, San Diego, CA). In this network, the nodes were lncRNAs or mRNAs, and when two nodes were connected by an edge, this indicated that they were coexpressed. The green and pink nodes in this network represented lncRNAs and mRNAs, respectively, whereas the solid lines and dotted lines showed positive and negative correlations, respectively.

### 2.7. Construction of the lncRNA-miRNA-mRNA Competing Endogenous RNA (ceRNA) Regulatory Network

The ceRNA hypothesizes that RNA transcripts can crosstalk by competing for common microRNAs (miRNAs), with miRNA response elements (MREs) being the foundation of this interaction [15]. These RNA transcripts have been termed ceRNAs and include pseudogene transcripts, lncRNAs, circRNAs, and mRNAs. Any RNA transcript with MREs can act as a ceRNA. These transcripts can compete for the regulation of the same MREs. To identify potential miRNA targets, a home-made miRNA target prediction software based on TargetScan and miRanda was used. By merging the common targeted miRNAs, we constructed a ceRNA network. Three conditions must exist for the ceRNA network to occur. First, the relative concentration of the ceRNAs and their miRNAs is clearly important; second, the effectiveness of a ceRNA depends on the number of miRNAs that it can “sponge”; third, not all of the MREs on ceRNAs are equal. Thus, we only selected the ceRNA-pair relations that passed this filtering.

In addition to measuring the number of common miRNAs, a hypergeometric test is executed for each ceRNA pair separately, which is defined by four parameters: (i) *N* is the total number of miRNAs used to predict targets, (ii) *K* is the number of miRNAs that interact with the chosen gene of interest, (iii) *n* is the number of miRNAs that interact with the candidate ceRNA of the chosen gene, and (iv) is the common miRNA number between the two genes [16]. The test calculates the *P* value using the following formula:

(1) $$\begin{array}{c} P=\overset{\min\left(K,n\right)}{\underset{i=c}{\sum }}\frac{\left(\begin{array}{c} K\\ i \end{array}\right)\left(\begin{array}{c} N-K\\ n-i \end{array}\right)}{\left(\begin{array}{c} N\\ n \end{array}\right)}. \end{array}$$

Red nodes represent miRNAs, light blue nodes represent mRNAs, and light green nodes represent lncRNAs. Edges with a T-shaped arrow represent directed relationships, and edges without arrows represent undirected relationships (ceRNA relationship).

### 2.8. Statistical Analysis

Statistical analyses were performed using SPSS 19.0. Fisher's exact test, Pearson correlation, and independent sample *t*-tests were used to identify significant differences, and a *P* of <0.05 was considered statistically significant. The false discovery rate (FDR) was calculated to correct the *P* value. A fold change > 2 and *P* < 0.05 were set as the threshold values to designate up- and downregulated lncRNAs and mRNAs.

## 3. Results

### 3.1. Demographic Characteristics

Detailed demographic data, smoking habits, comorbidities, and mean blood pressure levels of the five patients in the AAA group and the three patients in the control group in the screening stage are summarized in Table 1, whereas those of the 11 patients in the AAA group and the 12 patients in the control group in the validation stage are summarized in Table 2. Regarding the patients in the screening stage, the age (mean ± SD) of those in the AAA and control groups was 68.80 ± 10.33 and 43.33 ± 21.94 years, respectively (*P* > 0.05). The most common comorbidities among the enrolled patients are hypertension and coronary artery disease (CAD). The average blood pressure was higher in the AAA group than that in the control group (*P* < 0.05). No differences were noted in age, sex, BMI, smoking habit, and comorbidities between the two groups (*P* > 0.05). Regarding the patients in the validation stage, the age (mean ± SD) was 60.64 ± 10.51 and 52.83 ± 8.97 in the AAA and control groups, respectively. Of the patients in the AAA group, 90.90% were male, whereas of those in the control group, 75% were male (*P* > 0.05). There were no differences between the two groups in terms of age, sex, BMI, and smoking habit (*P* > 0.05). Hypertension was the most frequent comorbidity in the AAA group, while the most common comorbidity in the control group was CAD. However, the difference between the two groups was not statistically significant (*P* > 0.05). Furthermore, higher blood pressure levels (diastolic blood pressure) were observed in the AAA group than in the control group (*P* < 0.05).

### 3.2. Expression Profiles of lncRNAs and mRNAs in the Two Groups

**Figure 1:**
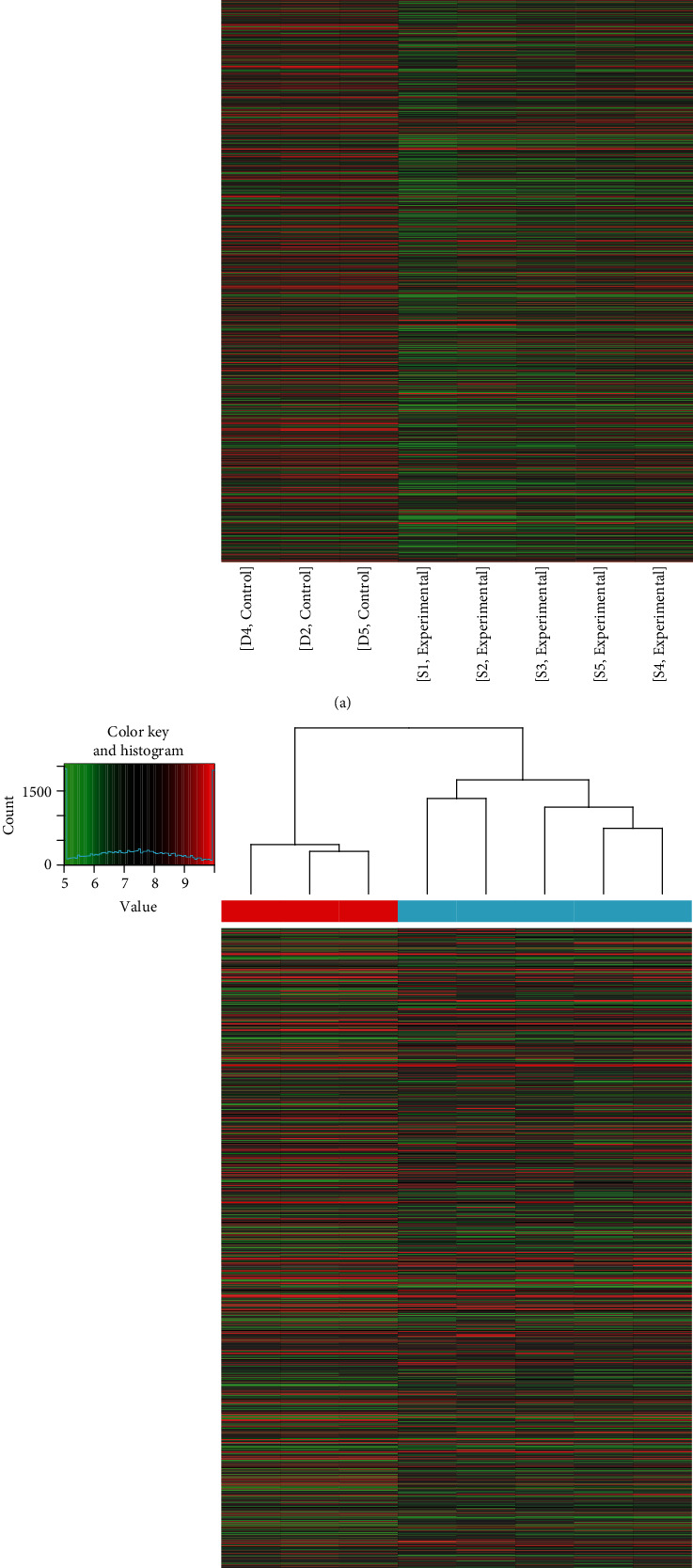
Hierarchical cluster of heatmaps for the differential expression of lncRNAs and mRNAs between the AAA and control groups. (a) Hierarchical cluster of heatmaps for the lncRNAs. (b) Hierarchical cluster of heatmaps for the mRNAs. Each column represents one sample and each row represents one mRNA or lncRNA. The relative expression levels of mRNAs and lncRNAs are depicted according to the color scale. Red indicates upregulation and green indicates downregulation. The three columns on the left represent the control samples and the five columns on the right represent the AAA samples. The differentially expressed mRNAs and lncRNAs are clearly self-segregated into clusters. AAA: abdominal aortic aneurysm; lncRNAs: long noncoding RNAs.

**Figure 2:**
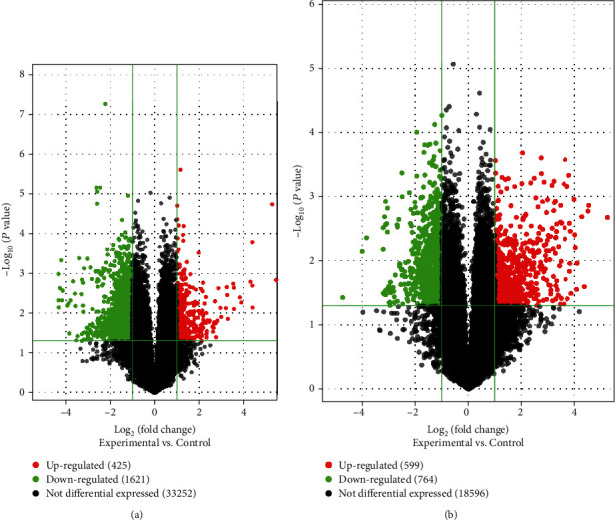
Volcano plots of all the lncRNAs and mRNAs detected in the AAA and control groups. The vertical dotted line at the *x*-axis delimits upregulation and downregulation. The red and green plots represent the significantly upregulated and downregulated genes, respectively (fold change > 2 and *P* < 0.05). The black plots represent no significant changes. (a) Volcano plots of the total 35298 lncRNAs. Of them, 425 are upregulated, 1621 are downregulated, and 33252 show no differential expression. (b) Volcano plots of the total 19959 mRNAs. Of them, 599 are upregulated, 764 are downregulated, and 18596 show no differential expression. AAA: abdominal aortic aneurysm; lncRNAs: long noncoding RNAs.

In total, 2046 lncRNAs and 1363 mRNAs were detected using the Arraystar lncRNA microarrays. Of these, 425 lncRNAs were upregulated and 1621 lncRNAs were downregulated, while 599 mRNAs were upregulated and 764 mRNAs were downregulated with a fold change > 2 and *P* < 0.05, when the AAA group was compared with the control group. Significantly differentially expressed lncRNAs and mRNAs were presented with hierarchical clusters of heatmaps (Figures 1(a) and 1(b)) and volcano plots (Figures 2(a) and 2(b)). Among these differentially expressed lncRNAs and mRNAs, the top 20 most significantly upregulated and downregulated lncRNAs are listed in Tables 3 and 4, respectively. In addition, the top 20 most significantly upregulated and downregulated mRNAs are shown in Tables 5 and 6.

### 3.3. Microarray-Based GO and KEGG Analyses

**Figure 3:**
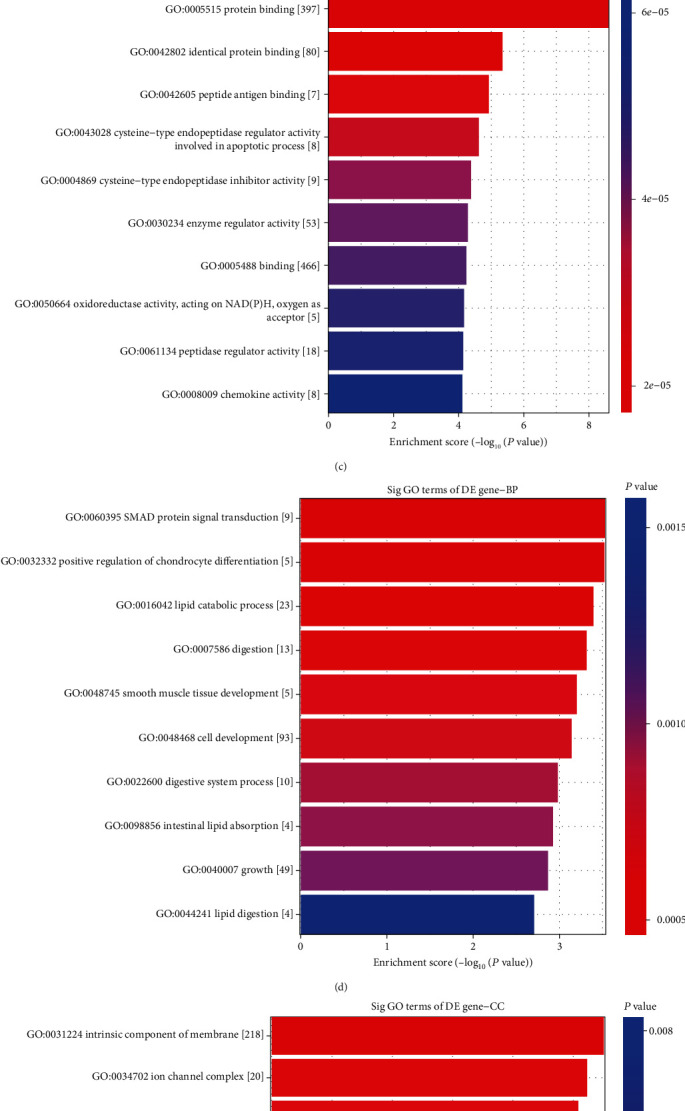
Gene Ontology analysis of differentially expressed mRNAs between the AAA and control groups. The top 10 GO terms from the upregulated mRNAs and the top 10 GO terms from the downregulated mRNAs between two groups. In upregulated mRNAs: (a) biological process, (b) cellular component, and (c) molecular function. In downregulated mRNAs: (d) biological process, (e) cellular component, and (f) molecular function. AAA: abdominal aortic aneurysm; GO: Gene Ontology.

The annotation of GO terms showed that upregulated mRNAs were involved in 1337 biological processes (BP), 154 cellular components (CC), and 142 molecular functions (MF) (Figures 3(a), 3(b), and 3(c)). The downregulated mRNAs were involved in 388 BP, 34 CC, and 58 MF (Figures 3(d), 3(e), and 3(f)). In the BP category, the highest enrichment scores of the GO term for upregulated mRNAs were immune response, while the highest for downregulated mRNAs were SMAD protein signal transduction. In the CC category, the most significant terms for upregulated mRNAs appeared in vesicles, and downregulated mRNAs appeared in the intrinsic component of the membrane. In the MF category, the most represented term for upregulated mRNAs was protein binding, and for downregulated mRNAs was 5′-nucleotidase activity.

**Figure 4:**
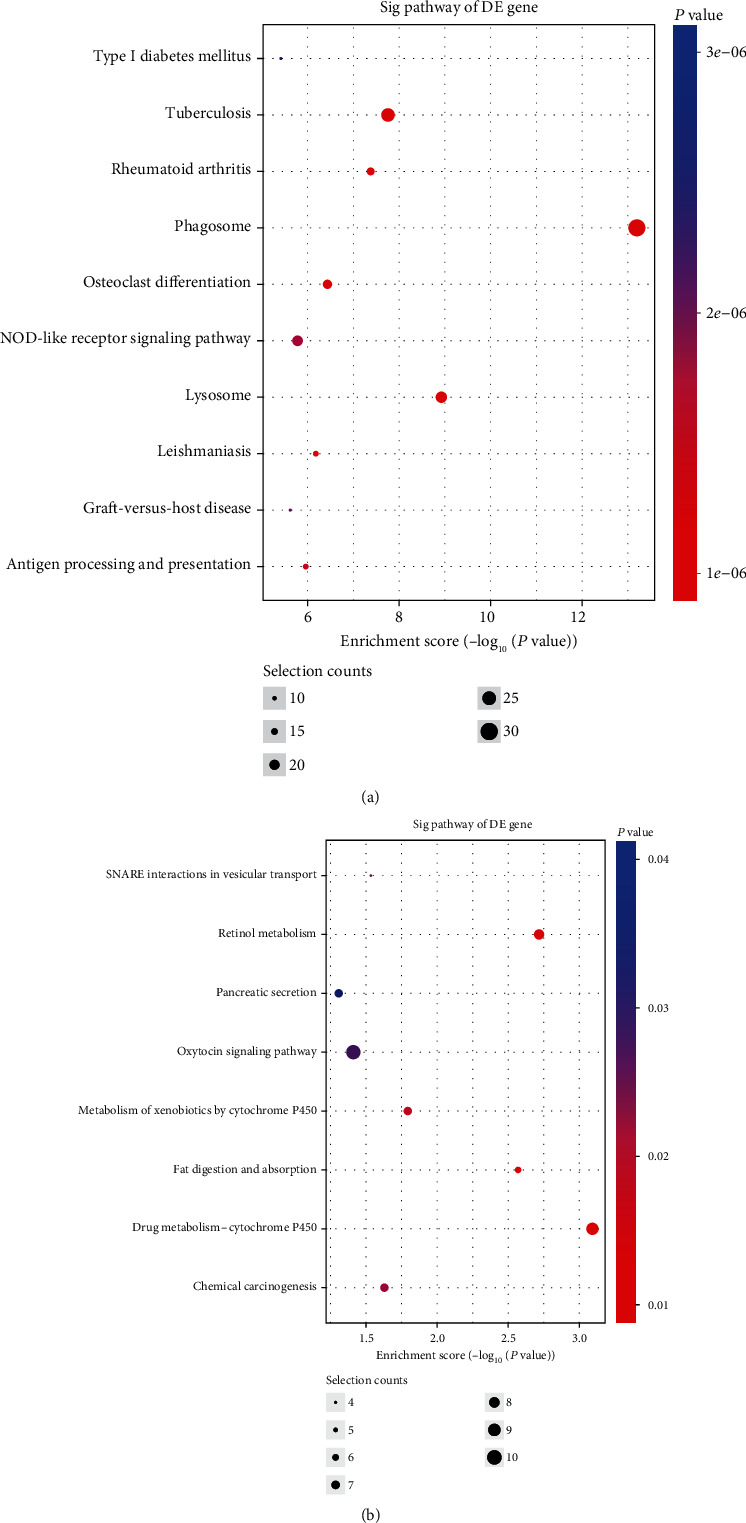
The top 18 pathways identified using KEGG pathway analysis of differently expressed mRNAs. KEGG analysis of the differentially expressed lncRNAs and mRNAs between the AAA and control groups. The top 18 significantly enriched KEGG pathway terms that correlated with the upregulated (a) and downregulated (b) genes between the two groups. AAA: abdominal aortic aneurysm; KEEG: Kyoto Encyclopedia of Genes and Genomes; lncRNAs: long noncoding RNAs.

KEGG pathway analysis revealed that the upregulated mRNAs were involved in 56 pathways, while the downregulated genes were involved in eight pathways. The highest enrichment score of pathways in upregulated mRNAs included the phagosome pathway and lysosome pathway (Figure 4(a)). For the downregulated mRNAs, the drug metabolism-cytochrome P450 pathway was the most enriched pathway (Figure 4(b)). Among all pathways, the NOD-like receptor (NLR) and NF-kappa B signaling pathways, cell adhesion molecules (CAMs), and the HIF-1 signaling pathway were closely related to the pathogenesis of AAA development. In addition, the top 10 upregulated pathways and the top eight downregulated pathways were compared with those in the control group.

### 3.4. Classification of Identified Genes

**Figure 5:**
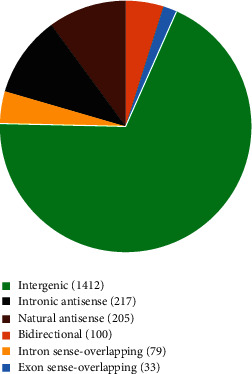
The classification of lncRNAs according to their correlations with protein-coding genes. All lncRNAs were classified to six categories including intergenic, intronic antisense, natural antisense, bidirectional, intron sense-overlapping, and exon sense-overlapping. The pie chart represents the numbers and distributions of the lncRNAs detected using the microarray. lncRNAs: long noncoding RNAs.

The association between lncRNAs and mRNAs was analyzed to identify putative functional relationships. The relationship between lncRNAs and mRNAs was categorized as intergenic, natural antisense, intronic antisense, exon sense-overlapping, bidirectional, and intron sense-overlapping. Among the differentially expressed lncRNAs, 1412 intergenic, 217 intronic antisense, 205 natural antisense, 100 bidirectional, 79 intron sense-overlapping, and 33 exon sense-overlapping lncRNAs were identified as shown in Figure 5.

### 3.5. Three Validated lncRNAs Were Downregulated in AAA Tissue

**Figure 6:**
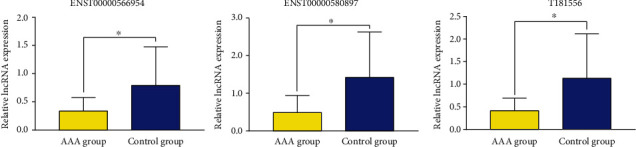
Validation of confirmed lncRNAs using qRT-PCR. The results showed that the relative expression levels of ENST00000566954, ENST00000580897, and T181556 were downregulated in the AAA group (*n* = 11) compared with those in the control group (*n* = 12). All reactions were repeated three times for each lncRNA. *β*-Actin was used as an internal control. ^∗^*P* < 0.05 AAAs vs. control. lncRNAs: long noncoding RNAs; AAA: abdominal aortic aneurysm.

To validate the significantly expressed lncRNAs detected using microarray analysis, 15 upregulated lncRNAs and 15 downregulated lncRNAs were selected for qRT-PCR validation in five AAA tissues and three normal aorta tissues. These lncRNAs were further validated in 11 versus 12 aortic samples. After strict validation, three novel lncRNAs (ENST00000566954, ENST00000580897, and T181556) were confirmed using qRT-PCR with three biological replicates between the AAA and control groups. The expression levels of ENST00000566954, ENST00000580897, and T181556 were downregulated in the AAA group compared with those in the control group, and the difference was statistically significant (Figure 6). The primer sequences were as follows: ENST00000566954, forward 5′-GCCCTCTTCTTCAAGGATGC-3′, reverse 5′-GCGGGCACATTTCACAGAT-3′; ENST00000580897, forward 5′-CCATCCAGGGTATTTCACAA-3′, reverse 5′-CTCCCATCTGTCTGCATCAA-3′; T181556, forward 5′-GAAAAGTATTTCCTTCCCTACAG-3′, reverse 5′-TTTAGATCCCAAAAATATGTGAG-3′; and *β*-actin, forward 5′-GTGGCCGAGGACTTTGATTG-3′, reverse 5′-CCTGTAACAACGCATCTCATATT-3′.

### 3.6. lncRNA-mRNA CNC

**Figure 7:**
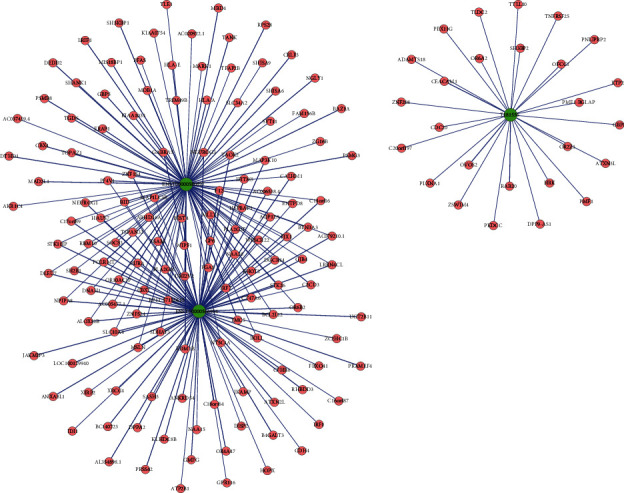
lncRNA-mRNA coexpression network of the differentially expressed lncRNAs and mRNAs between the two groups. The green and pink nodes represent the lncRNAs and mRNAs, respectively. The node size represents the degree of centrality of the gene in the network, defined as the link numbers of the node. The solid lines show a positive correlation; dotted lines show a negative correlation. lncRNAs: long noncoding RNAs.

To determine the relationship and the potential modulating mechanism between the aberrantly expressed mRNAs and lncRNAs, a lncRNA-mRNA CNC was constructed. Three strictly validated lncRNAs were used to build the network. Based on the criteria including a PCC not less than 0.97, *P* value ≤ 0.05, and FDR ≤ 1 between mRNAs and lncRNAs, the network containing the three aberrantly expressed lncRNAs and the 165 most highly relevant dysregulated mRNAs is shown in Figure 7. This CNC was composed of 120 positive and 109 negative interactions. These genes were suggested to play critical roles in the CNC. Further details about the lncRNA-mRNA network analysis are shown in Table 7.

### 3.7. Construction of the lncRNA-miRNA-mRNA ceRNA Regulatory Network

**Figure 8:**
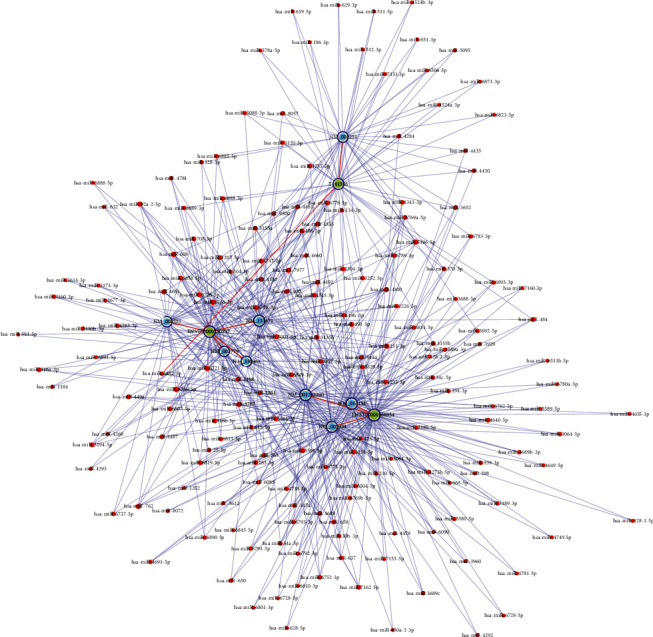
The lncRNA-miRNA-mRNA ceRNA regulatory network constructed based on three confirmed lncRNAs in the AAA and control groups. The nodes highlighted in red represent microRNAs, the nodes highlighted in light blue represent protein-coding RNAs, and those highlighted in light green represent lncRNAs. Edges with a T-shaped arrow represent directed relationships, while those without an arrow represent undirected relationships (ceRNA relationships). lncRNAs: long noncoding RNAs; miRNA: microRNA; ceRNA: competing endogenous RNA; AAA: abdominal aortic aneurysm.

To better understand the regulatory mechanism of validated lncRNAs in AAA development, a ceRNA network was constructed. It is helpful to elucidate the interaction between these differentially expressed lncRNAs and differentially expressed miRNAs, in addition to determining the interaction between miRNAs and mRNAs, as shown in Figure 8.

## 4. Discussion

AAA, characterized by chronic degenerative processes of the abdominal aorta, is one of the leading causes of cardiovascular death [17]. AAA patients who are below the currently accepted threshold for surgical intervention have a risk of spontaneous rupture during follow-up owing to the absence of effective drugs to limit the growth of small AAAs [18]. One of the main reasons is that the specific molecular mechanism involved in AAA formation is still unclear. Microarray-based screening approaches are useful for identifying novel diagnostic and therapeutic targets. For instance, Yang et al. [19] reported that lnc-ARG was identified in samples from three AAA patients and three control subjects using microarray analysis, revealing that new lncRNA candidates are related to the pathogenesis of AAA. Moreover, numerous lncRNAs have been proved to have important regulatory roles in cardiovascular diseases [20]. Therefore, we used lncRNA microarray technology to analyze the lncRNA expression profile of the AAA and control samples.

Our results showed that 2046 lncRNAs (425 upregulated and 1621 downregulated lncRNAs) and 1363 mRNAs (599 upregulated and 764 downregulated mRNAs) were identified between the AAA and control groups with a fold change cutoff of 2 (*P* < 0.05) using the Arraystar lncRNA microarrays. Interestingly, annotation of GO terms revealed that the most differentially expressed genes from the two groups were mostly associated with SMAD protein signal transduction and immune response. Tan et al. [21] documented that SMAD3 deficiency resulted in defective aortic biomechanics and physiological functions, which caused weakening of the aortic wall. Li et al. [22] reported that the immune system participates in the regulation of the AAA pathological process and that it has a significant effect on AAA-related inflammatory reactions. Our annotation of GO terms was in accordance with previous reports.

KEGG pathway analysis showed that phagosomes are the most enriched pathways in upregulated genes, and drug metabolism-cytochrome P450 is the most enriched pathway in downregulated genes. Among the significantly enriched pathways, the NOD-like receptor (NLR) pathway, cell adhesion molecule (CAM), and the NF-kappa B (NF-*κ*B) signaling pathway are potentially closely related to AAA pathogenesis [23–25]. It was considered that these lncRNAs might participate in the biological process of SMAD protein signal transduction and immune response in AAA progression via mediating the above significantly enriched pathways.

The lncRNAs identified using microarray analysis were validated in a large number of samples to improve the reliability of the microarray data. To determine the potential downstream targets of lncRNAs, a CNC and a ceRNA network were constructed. CNC analysis predicted that SOCS3 may bind with ENST00000580897, the downstream target with the highest correlation (PCC = 0.999). A relevant study showed that overexpression of SOCS3 in bone marrow-derived cells significantly increased aneurysm severity (*P* = 0.04) demonstrating that STAT3/SOCS3 signaling in bone marrow-derived cells contributes to AAA development [26]. This indicates that the results of CNC analysis are reliable. The ceRNA network predicted that T181556 could directly bind to miR-212-5p and miR-145. Tian et al. [27] reported that LINC00473 participates in AAA development by regulating the miR-212-5p/BASP1 pathway, suggesting that LINC00473 is a promising target for AAA therapy. Lin et al. [28] revealed that downregulation of lncRNA Sox2ot suppressed the expression of Egr1 by regulating miR-145, highlighting a theoretical basis for AAA treatment. Furthermore, this result indicates that lncRNAs can regulate the biological impact of miRNAs within disease onset and progression by functioning as miRNA sponges.

Emerging evidence has demonstrated the important role of lncRNAs in AAA formation, including VSMC proliferation, VSMC apoptosis, and phenotypic switching, such as that of lncRNA-p21, and that lncRNAs mediate SMC survival and macrophage activity in the atherosclerotic process [29]. lncRNA NEAT1 regulates the phenotypic switching of VSMCs by repressing smooth muscle-contractile gene expression through an epigenetic regulatory mechanism [30]. In addition, H19 is one of the widely studied lncRNAs in various diseases. Li et al. [31] reported that a mouse lncRNA microarray analysis using two murine AAA models identified a high upregulation of lncRNA H19, indicating that it is a novel regulator of SMC survival in AAA development. Thus, inhibition of H19 expression might be a novel molecular targeted therapeutic strategy against AAA development. lncRNA GAS5 overexpression in SMCs induced apoptosis and repressed proliferation. Furthermore, GAS5 acted as an miR-21 sponge releasing phosphatase and tensin homolog from repression, thereby promoting AAA formation in two murine AAA models [32].

Interestingly, as lncRNAs have high tissue specificity, they have the potential to become favorable diagnostic and prognostic biomarkers for cardiovascular diseases. lncRNA XLOC_009167 was identified using lncRNA microarray and confirmed to be a circulating biomarker using qRT-PCR in the whole blood of lung cancer patients. XLOC_009167 serves as a diagnostic biomarker that distinguishes lung cancer from benign lung disease [33]. Li et al. [34] reported that two novel lncRNA biomarkers, ENST00000444488.1 and uc010yfd.1, were identified using microarray analysis of transcriptome-wide lncRNA and mRNA expression profiles of peripheral blood mononuclear cells (PBMCs) of 93 CAD patients and 48 healthy controls, indicating that those lncRNAs had the best value for distinguishing CAD patients from healthy controls. Our PCR validation assay showed that the expression levels of three validated lncRNAs were significantly lower in the AAA group than in the control group, indicating that these lncRNAs may serve as diagnostic biomarkers to predict the growth rate of aneurysms and risk of rupture. However, a large blood sample size is needed to confirm their diagnostic and prognostic value in clinical applications.

Some limitations of our study merit consideration. Frist, since all the patients enrolled in this study were recruited from a single medical center, an enlarged multicenter sample cohort study is needed to further confirm the accuracy of our microarray results and diagnostic value of candidate lncRNAs. Second, the underlying molecular mechanism of how these lncRNAs regulate AAA progression needs to be investigated in future in vitro and in vivo studies.

## 5. Conclusions

In conclusion, we revealed the lncRNA and mRNA expression profiles of AAA patients and control individuals using microarray analysis. Three novel lncRNAs were successfully identified as diagnostic biomarkers. Moreover, our lncRNA-mRNA and ceRNA network analyses provide potential therapeutic targets for AAA.

## Figures and Tables

**Table 1:** Clinical characteristics of AAA patients and controls in screening stage.

Characteristics	AAA (*n* = 5)	Controls (*n* = 3)	*P* value
Age (years)	68.80 ± 10.33	43.33 ± 21.94	0.062
Gender (male)	5 (5)	3 (1)	0.107
BMI	26.06 ± 4.19	25.69 ± 1.88	0.89
Smoking habit	5 (3)	3 (1)	0.465
Comorbidities			0.781
Hypertension	4	1	
Diabetes mellitus	0	0	
Dyslipidemia	1	1	
CAD	2	1	
COPD	1	0	
SBP (mmHg)	150 ± 11.40	119 ± 15.59	0.017
DBP (mmHg)	90.20 ± 8.67	63.67 ± 8.39	0.005

Those clinical data are presented as the mean ± standard deviation. *P* < 0.05 showed difference was statistically significant between the AAA and control groups. CAD: Coronary artery disease; COPD: Chronic obstructive pulmonary Disease; SBP: Systolic blood pressure; DBP: Diastolic blood pressure.

**Table 2:** Clinical characteristics of AAA patients and controls in validation stage.

Characteristics	AAA (*n* = 11)	Controls (*n* = 12)	*P* value
Age (years)	60.64 ± 10.51	52.83 ± 8.97	0.081
Gender (male)	11 (10)	12 (9)	0.315
BMI	24.19 ± 3.71	25.88 ± 4.17	0.275
Smoking habit	11 (5)	12 (2)	0.855
Comorbidities			0.165
Hypertension	9	3	
Diabetes mellitus	2	1	
Dyslipidemia	0	1	
CAD	2	5	
COPD	0	1	
SBP (mmHg)	143.73 ± 15.64	133.33 ± 11.17	0.079
DBP (mmHg)	90.36 ± 14.62	74.75 ± 7.24	0.003

*P* < 0.05 showed difference was statistically significant between the AAA and control groups. BMI: Body mass index; CAD: Coronary artery disease; COPD: Chronic obstructive pulmonary disease; SBP: Systolic blood pressure; DBP: Diastolic blood pressure.

**Table 3:** The top 20 differentially upregulated lncRNAs.

lncRNA seqname	Probe name	*P* value	FC	Regulation	Chr	Strand
ENST00000489312	ASHGV40037100	1.45909*E* − 02	43.941	Up	Chr4	−
NR_024376	ASHGV40052194	2.63232*E* − 02	43.94	Up	Chr9	−
T070519	ASHGV40058117	1.61205*E* − 02	43.941	Up	Chr11	−
NR_037938	ASHGV40044103	2.16161*E* − 02	43.941	Up	Chr6	−
NR_121585	ASHGV40052592	9.31431*E* − 03	43.941	Up	Chr9	+
TCONS_00016231	ASHGV40053646	2.94133*E* − 02	43.941	Up	Chr9	+
ENST00000446590	ASHGV40001027	2.75747*E* − 03	43.941	Up	Chr2	−
T049579	ASHGV40006291	1.05401*E* − 03	43.941	Up	Chr10	+
T201134	ASHGV40027570	1.00830*E* − 02	43.941	Up	Chr2	−
NR_126330	ASHGV40004795	1.02576*E* − 02	43.941	Up	Chr16	−
ENST00000558101	ASHGV40002158	2.07440*E* − 02	43.941	Up	Chr15	+
T285383	ASHGV40042013	4.13377*E* − 02	43.941	Up	Chr5	+
T308480	ASHGV40045426	4.03730*E* − 02	43.941	Up	Chr6	+
T089165	ASHGV40058187	1.76097*E* − 05	43.941	Up	Chr12	+
ENST00000568457	ASHGV40046457	7.71652*E* − 04	43.941	Up	Chr17	−
T060416	ASHGV40007110	3.69880*E* − 03	38.929	Up	Chr11	−
T198099	ASHGV40029430	1.53891*E* − 02	21.088	Up	Chr2	+
ENST00000544089	ASHGV40011648	2.87345*E* − 03	20.943	Up	Chr12	+
NR_002812	ASHGV40044859	3.72950*E* − 02	20.943	Up	Chr6	+
T164886	ASHGV40023628	8.90619*E* − 03	20.943	Up	Chr18	+

Note: seqname: the name of lncRNAs; *P* value: *P* values were calculated by the unpaired *t*-test; fold change (FC): the absolute ratio of normalized intensities between the AAA and control groups; regulation: it depicts which group has greater or lower intensity values than another group; Chr: chromosome number from which the lncRNAs were transcribed; strand: “+” represented the sense strand of the chromosome, and “−” represented the antisense strand of the chromosome.

**Table 4:** The top 20 differentially downregulated lncRNAs.

lncRNA seqname	Probe name	*P* value	FC	Regulation	Chr	Strand
T280062	ASHGV40040040	1.04530*E* − 03	19.868	Down	Chr5	−
ENST00000597337	ASHGV40056562	4.73312*E* − 03	19.790	Down	Chr19	−
T379563	ASHGV40059212	7.12996*E* − 03	19.690	Down	ChrX	−
ENST00000445280	ASHGV40057551	2.33336*E* − 03	19.034	Down	Chr6	−
T041698	ASHGV40005622	4.68507*E* − 04	18.200	Down	Chr10	+
NR_110838	ASHGV40037966	4.72916*E* − 03	17.751	Down	Chr4	−
TCONS_00019584	ASHGV40006697	2.71932*E* − 03	17.404	Down	Chr11	−
ENST00000428188	ASHGV40027175	6.46729*E* − 03	15.067	Down	Chr2	−
T256902	ASHGV40035410	5.69074*E* − 03	14.540	Down	Chr3	−
NR_027143	ASHGV40056682	3.20755*E* − 02	14.117	Down	Chr2	−
TCONS_00014675	ASHGV40059042	4.76868*E* − 03	13.605	Down	Chr8	+
T343400	ASHGV40049251	1.59113*E* − 03	12.591	Down	Chr8	−
T181556	ASHGV40024838	3.22298*E* − 03	11.865	Down	Chr19	−
uc.243+	ASHGV40060161	6.61118*E* − 03	11.667	Down	Chr8	+
ENST00000559321	ASHGV40017346	4.78557*E* − 02	11.389	Down	Chr15	+
NR_027142	ASHGV40027253	3.73950*E* − 02	10.946	Down	Chr2	−
T314169	ASHGV40045879	4.16561*E* − 04	10.386	Down	Chr6	+
T380669	ASHGV40054465	3.31702*E* − 03	9.681	Down	ChrX	−
ENST00000590513	ASHGV40056388	3.33256*E* − 02	9.592	Down	Chr17	−
ENST00000561471	ASHGV40002223	4.97655*E* − 02	9.577	Down	Chr16	−

Note: FC: fold change.

**Table 5:** The top 20 differentially upregulated mRNAs.

mRNA seqname	Probe name	*P* value	FC	Regulation	Chr	Strand
NM_005623	ASHGV40021590	3.84884*E* − 02	37.689	Up	Chr17	+
NM_020198	ASHGV40020691	4.27559*E* − 02	23.178	Up	Chr17	−
NM_019029	ASHGV40046319	3.63187*E* − 03	22.732	Up	Chr7	−
NM_003816	ASHGV40050544	4.25717*E* − 02	20.735	Up	Chr8	+
NM_001912	ASHGV40053068	1.18592*E* − 02	19.413	Up	Chr9	+
NM_014391	ASHGV40055816	6.25422*E* − 03	17.168	Up	Chr10	−
NM_022154	ASHGV40037821	4.69229*E* − 04	16.922	Up	Chr4	−
NM_006927	ASHGV40018205	4.84306*E* − 03	16.432	Up	Chr16	−
NM_004388	ASHGV40009210	1.67921*E* − 02	16.176	Up	Chr1	−
NM_001278795	ASHGV40005594	1.01539*E* − 02	15.887	Up	Chr10	+
NM_001165	ASHGV40009065	2.60247*E* − 03	14.665	Up	Chr11	+
NM_130899	ASHGV40041056	4.92976*E* − 03	14.449	Up	Chr5	−
NM_080596	ASHGV40044765	2.97741*E* − 02	14.285	Up	Chr6	+
NM_033129	ASHGV40030434	1.16722*E* − 03	14.186	Up	Chr20	−
NM_002950	ASHGV40034879	4.63507*E* − 02	14.073	Up	Chr3	−
NM_138610	ASHGV40040832	1.78745*E* − 02	13.837	Up	Chr5	−
NM_005522	ASHGV40046294	1.50570*E* − 02	13.241	Up	Chr7	−
NM_006936	ASHGV40032377	2.34477*E* − 02	13.176	Up	Chr21	−
NM_018184	ASHGV40035555	2.37979*E* − 02	12.629	Up	Chr3	+
NM_174899	ASHGV40030233	1.35807*E* − 02	12.512	Up	Chr2	+

Note: FC: fold change.

**Table 6:** The top 20 differentially downregulated mRNAs.

mRNA seqname	Probe name	*P* value	FC	Regulation	Chr	Strand
NM_019005	ASHGV40047513	1.44230*E* − 02	26.542	Down	Chr7	+
NM_005934	ASHGV40023784	4.05184*E* − 02	15.973	Down	Chr19	−
NM_145252	ASHGV40018598	3.24925*E* − 02	14.266	Down	Chr16	+
ENST00000399910	ASHGV40014780	1.18588*E* − 02	9.253	Down	Chr14	+
NM_004877	ASHGV40024375	1.06567*E* − 02	9.247	Down	Chr19	−
NM_001205	ASHGV40042738	1.80411*E* − 02	9.229	Down	Chr5	+
NM_007203	ASHGV40053297	2.45544*E* − 02	8.944	Down	Chr9	+
NM_198564	ASHGV40034459	1.66168*E* − 02	8.906	Down	chr13	−
NM_001242901	ASHGV40025050	1.51714*E* − 03	8.703	Down	Chr19	+
NM_052874	ASHGV40017864	1.32087*E* − 02	8.700	Down	chr16	−
uc011dig.1	ASHGV40000054	7.13034*E* − 03	8.678	Down	Chr6	−
NM_006414	ASHGV40005351	4.85080*E* − 02	8.566	Down	Chr10	+
NM_003215	ASHGV40037479	1.41224*E* − 03	8.432	Down	Chr4	−
NM_005361	ASHGV40057658	2.77734*E* − 02	8.305	Down	ChrX	+
NM_016619	ASHGV40037718	1.95059*E* − 02	8.267	Down	Chr4	−
NM_007081	ASHGV40056879	4.42568*E* − 03	8.185	Down	Chr22	−
NM_005500	ASHGV40025840	2.18217*E* − 02	7.965	Down	Chr19	+
NM_001145204	ASHGV40018716	1.22095*E* − 02	7.895	Down	Chr16	+
NM_176820	ASHGV40024835	9.32347*E* − 03	7.789	Down	Chr19	−
NM_001310219	ASHGV40019854	4.61601*E* − 03	7.787	Down	Chr17	−

Note: FC: fold change.

**Table 7:** lncRNA-mRNA coexpression network analysis between the confirmed lncRNAs and mRNAs.

lncRNA seqname	Target mRNA gene symbol	Correlation	*P* value	FDR
ENST00000566954	WIPF1	0.994	4.94602*E* − 07	8.08971*E* − 05
	GP6	0.995	3.76023*E* − 07	6.68503*E* − 05
	POLRMT	-0.995	3.48896*E* − 07	6.68503*E* − 05
	LRRN4CL	-0.992	1.12833*E* − 06	1.39810*E* − 04
	PLA2G2F	-0.997	7.90082*E* − 08	2.30760*E* − 05
	RBM10	-0.993	9.00992*E* − 07	1.15130*E* − 05
	C11orf16	-0.995	3.63929*E* − 07	6.68503*E* − 05

ENST00000580897	C2CD3	-0.995	2.52021*E* − 07	6.06185*E* − 05
	SDHAF3	0.996	1.51514*E* − 07	3.87212*E* − 05
	NT5C1A	0.995	2.81966*E* − 07	6.40533*E* − 05
	DGCR14	0.997	6.94397*E* − 08	2.18415*E* − 05
	F12	0.999	6.96601*E* − 09	4.74734*E* − 06
	SH2B1	0.997	5.74742*E* − 08	2.13647*E* − 05
	PGA5	0.994	6.64443*E* − 07	9.70324*E* − 05
	GJB4	0.999	6.28164*E* − 13	2.56856*E* − 09
	DNAH1	0.998	1.46018*E* − 08	7.46336*E* − 06
	MAP3K10	0.993	7.86261*E* − 07	1.10216*E* − 04
	HAUS7	0.999	1.16229*E* − 10	2.376297*E* − 07
	TSPAN32	0.997	4.71800*E* − 08	1.92919*E* − 05
	SOCS3	0.999	5.24868*E* − 09	4.29236*E* − 06
	ESAM	0.993	8.35582*E* − 07	1.10216*E* − 04
	STK11IP	0.994	5.76505*E* − 07	8.80655*E* − 05
	DLEU7	0.995	3.26488*E* − 07	6.68503*E* − 05
	C17orf89	0.994	5.81504*E* − 07	8.80655*E* − 05
	RBM10	0.995	3.69676*E* − 07	6.68503*E* − 05
	NPIPA8	0.997	6.81732*E* − 08	2.18415*E* − 05
	TAOK2	0.999	9.00736*E* − 10	1.22770*E* − 06
	AC079210.1	0.993	8.29330*E* − 07	1.10216*E* − 04
	NEUROG1	0.997	9.99007*E* − 08	2.72329*E* − 05
	AQP12A	0.999	1.80042*E* − 09	1.84048*E* − 06
	C11orf16	0.995	3.97800*E* − 07	6.77752*E* − 05
	FJX1	0.998	9.83848*E* − 09	5.74708*E* − 06

T181556	DPP9-AS1	0.992	1.28236*E* − 06	1.54223*E* − 04

Note: lncRNA-mRNA network was constructed based on PCC≧0.8; *P* value ≤ 0.05 and FDR ≤ 1 between the three validated lncRNAs and 165 most highly relevant dysregulated mRNAs. FDR: false discovery rate.

## Data Availability

The data used to support the findings of this study are available from the corresponding author upon request.
